# Cardiometabolic Effects of *Nigella sativa* in Postmenopausal Women with Hypertension: A Prospective, Observational, Pilot Study

**DOI:** 10.3390/nu17060985

**Published:** 2025-03-11

**Authors:** Barbara Pala, Giulia Nardoianni, Paola Gualtieri, Giulia Frank, Marco Alfonso Perrone, Laura Di Renzo, Giuliano Tocci

**Affiliations:** 1 Division of Cardiology, Department of Clinical and Molecular Medicine, University of Rome Sapienza, Sant’Andrea Hospital 1035/1039, 00189 Rome, Italygiuliano.tocci@uniroma1.it (G.T.); 2 PhD School of Applied Medical-Surgical Sciences, University of Rome Tor Vergata, Via Montpellier 1, 00133 Rome, Italy; 3 Section of Clinical Nutrition and Nutrigenomics, Department of Biomedicine and Prevention, University of Rome “Tor Vergata”, Via Montpellier 1, 00133 Rome, Italy; laura.di.renzo@uniroma2.it; 4 School of Specialization in Food Science, University of Rome Tor Vergata, 00133 Rome, Italy; 5 Division of Cardiology and CardioLab, Department of Clinical Sciences and Translational Medicine, University of Rome Tor Vergata, 00133 Rome, Italy

**Keywords:** menopause, hypertension, *Nigella sativa*, nutraceutics, cardiovascular prevention

## Abstract

**Background**: Hypertension is a prevalent condition, impacting a significant amount of general population and contributing prominently to global mortality. Increasing attention has been directed towards phytotherapy products as potential complementary or alternative therapies for hypertension prevention and treatment. Among these, *Nigella sativa* (NS) has shown encouraging effects in improving cardiovascular parameters. This study aimed to evaluate the efficacy of NS supplementation in reducing seated office systolic blood pressure (BP) in postmenopausal women. We also explored the dose-dependent effects of this intervention on BP levels and metabolic parameters. **Materials and Methods**: We conducted an observational pilot study including 52 women, who were stratified into two active groups (*n* = 32) receiving two different dosages of NS (*n* = 16, age 54.2 ± 2.3 at 400 mg/day and *n* = 16, age 52.3 ± 2.4 at 800 mg/day) and a control group (*n* = 20, age 53.9 ± 3.0). Participants were evaluated at baseline (T0), at 4 (T1) and 8 weeks (T2) for office brachial and central BP, heart rate (HR), lipid profile, body weight, and menopausal symptoms. **Results**: NS supplementation significantly reduced office systolic and diastolic BP in a dose-dependent manner (*p* < 0.01), with more pronounced reductions at 800 mg/day. Improvements in climacteric symptoms and reduced HR were observed as early as T1, while metabolic parameters, including lipid profile and weight, showed significant changes at T2. Notably, the 800 mg/day dosage group also experienced significant reductions in weight and body mass index. Younger age, more recent menopausal transition, and elevated baseline HR were identified as predictors of a better response to treatment. **Conclusions**: NS supplementation demonstrates significant dose-dependent benefits in reducing office BP and improving metabolic parameters. These findings support the role of NS as an effective complementary therapy in hypertension management in postmenopausal women.

## 1. Introduction

Hypertension is an asymptomatic condition, often referred to as a “silent killer”, due to its significant role in the development of acute cardiovascular diseases (CVD), such as myocardial infarction, stroke, chronic kidney disease (CKD), and heart failure. Its global prevalence represents a critical public health challenge, significantly contributing to morbidity and mortality at global level [1,2]. The World Health Organization highlights the alarming prevalence of this condition, which, if untreated, may lead to profound health and economic consequences, particularly in women [3]. Lifestyle modifications, including a diet rich in fruits and vegetables, reduced salt and alcohol intake, regular exercise, weight management, and smoking cessation, are recommended as initial strategy to reduce high blood pressure (BP) in all patients suffering with hypertension [4]. If lifestyle modifications alone do not achieve the recommended BP therapeutic targets, pharmacological drug interventions have to be started. Angiotensin-converting enzyme inhibitors (ACEIs), angiotensin II receptor blockers (ARBs), calcium channel blockers (CCBs), and diuretics have proven to be effective in managing high BP and reducing CVD risk [5]. Despite their efficacy, however, these therapies are frequently associated with adverse effects and limited long-term adherence, prompting patients and clinicians to explore alternative or complementary treatments [2].

In recent years, the use of natural and plant-based therapeutics has gained traction as a potential adjunctive strategy for managing hypertension and associated cardiovascular conditions. Among these, herbal products such as green tea, garlic, berberine, ginseng, *Nigella sativa*, cinnamon, ginger, and pomegranate have been widely studied for their bioactive properties and synergistic effects when combined with standard antihypertensive medications [1,6]. In particular, *Nigella sativa* (NS), commonly known as “black seed” or “black cumin”, has demonstrated a wide spectrum of pharmacological activities. Its primary bioactive compound, thymoquinone (TQ), has been shown to exert antioxidant, anti-inflammatory, antihypertensive, hepatoprotective, glucose lowering, and lipid-lowering effects, making NS a promising candidate for addressing complex, multifactorial conditions, like hypertension [6,7].

Recent clinical studies demonstrated the efficacy of NS in BP regulation. Indeed, NS exerts its antihypertensive effects through multiple pathways, including modulation of the renin–angiotensin–aldosterone system (RAAS), inhibition of sympathetic nervous system (SNS) activity, and enhancement of nitric oxide bioavailability, all of which improve endothelial function and reduce peripheral vascular resistances [4,7]. Additionally, NS may counteract the effects of angiotensin II, leading to reductions in left ventricular end-diastolic pressure, mean arterial pressure, and heart rate.

The mechanism by which *Nigella sativa* appears to exert its antihypertensive action involves the inhibition of angiotensin-converting enzyme (ACE), resulting in reduced blood pressure in L-NAME-induced hypertensive rats. This effect is further enhanced when *N. sativa* is administered alongside losartan, leading to a more pronounced decrease in blood pressure [8]. Additionally, increased cardiac heme oxygenase-1 (HO-1) activity associated with *N. sativa* reduces angiotensin II-induced inflammation and NADPH oxidase-mediated oxidative stress, which collectively contribute to the attenuation of hypertension in this model [2]. Its antioxidant properties, mediated by increased activity of enzymes such as superoxide dismutase and catalase, while lowering malondialdehyde levels, further contribute to its cardioprotective effects [1,9]. Notably, NS has demonstrated a favorable safety profile, with minimal adverse effects reported in diverse populations [6,7].

Despite these promising findings, gaps persist in research, particularly regarding the inclusion of women in clinical trials on hypertension. Women, especially those who are postmenopausal, remain underrepresented despite their distinct CVD risk profiles [3]. Hormonal changes during menopause, coupled with increased arterial stiffness, significantly heighten their susceptibility to hypertension and related complications [7]. Addressing this disparity is critical for developing gender-specific approaches to CVD prevention and treatment. In this context, several studies have explored the impact of NS on menopausal conditions, particularly in the presence of metabolic syndrome. The supplementation demonstrated a significant reduction in fasting plasma glucose (FPG), total cholesterol (TOT-C), low-density lipoprotein cholesterol (LDL-C), and triglycerides (TG) [10]. Furthermore, recent evidence underscores the link between climacteric syndrome and elevated CVD risk, highlighting the need for targeted interventions during menopause. While NS shows promise as a natural agent for mitigating these risks, clinical studies evaluating its efficacy in this specific population remain limited [7]. Comprehensive and well-designed studies are essential to fully elucidate the therapeutic potential of NS.

To contribute to this emerging area of research, the present pilot study evaluated the effects of NS supplementation at varying doses in reducing office systolic/diastolic BP levels at both brachial and central (aortic) levels in hypertensive postmenopausal women, alongside their standard antihypertensive therapy. By focusing on this clinically vulnerable and frequently underrepresented demographic, the study aims to advance the understanding of natural therapeutics in cardiovascular health and offer insights into the role of NS as an adjunctive therapy for essential uncomplicated hypertension in postmenopausal women.

## 2. Materials and Methods

### 2.1. Study Design

This observational pilot study was conducted between March 2024 and June 2024 at the Hypertension Unit, Division of Cardiology, Sant’Andrea Hospital, Sapienza University of Rome and Section of Clinical Nutrition and Nutrigenomics, Department of Biomedicine and Prevention of the University of Rome Tor Vergata. The study included postmenopausal women who were consecutively evaluated for hypertension screening and global cardiovascular risk stratification, including assessment of hypertension-mediated organ damage (HMOD).

Eligible participants had to meet the following inclusion criteria: (1) female sex; (2) postmenopausal status for at least two years; (3) confirmed diagnosis of uncomplicated essential hypertension (systolic blood pressure > 140 mmHg and diastolic blood pressure > 90 mmHg, or treated with anti-hypertensive drugs), without a history of cerebral (prior stroke), vascular (carotid plague), or renal disease (eGRF < 60 mL/min); (4) provision of signed written informed consent. Participants were excluded if they were undergoing hormonal replacement therapy, had a history of active cancer within the past five years, exhibited any mental health disorders (including premenopausal depression documented with medical certification), had drug or alcohol addiction, or were unwilling to attend clinical evaluations at predefined time intervals.

Participants were selected from the institutional database based on suboptimal BP control despite antihypertensive therapies, defined as having high-normal BP (systolic/diastolic BP 130–139/85–89 mmHg) or grade 1 hypertension (systolic/diastolic BP 140–159/90–99 mmHg). Patients were stratified into three groups: (a) case group 1: participants received a specific dose of NS supplementation (400 mg); (b) case group 2: participants received a higher dose of NS supplementation (400 mg, twice a day 2); (c) control group: participants received no supplementation and only attended monthly follow-up visits. In all groups, background standard antihypertensive therapies were maintained.

Group assignments were agreed upon in collaboration with the patients, given the observational nature of the study. Participants were instructed to maintain their usual medications and lifestyle habits, including dietary patterns (salt, fat, and vegetable consumption), smoking habits, physical activity levels, and any ongoing supplementation use. No additional dietary or lifestyle interventions were introduced during the study period.

We employed the pharmaceutical formulation NISATOL^®^, a soft gel capsule provided by PharmExtracta S.p.A., Piacenza, Italy. Each capsule contains 400 mg of *Nigella sativa* seed oil, 3.3 mg of Vitamin E, and additional components such as vegetable gelatin, glycerol, and water.

### 2.2. Study Procedure

Comprehensive demographic and clinical data for all participants were collected. Anthropometric assessments included height, weight, body mass index (BMI), and waist-to-hip ratio (WHR) were performed at baseline (T0), 4 weeks (T1), and 8 weeks (T2). BMI was calculated as weight in kilograms divided by the square of height in meters, with categorization based on World Health Organization (WHO) guidelines [11]. WHR was calculated, with a value exceeding 0.85 indicating elevated cardiovascular risk in women [12].

Office systolic and diastolic BP and central (aortic) BP were measured non-invasively by using a validated, oscillometric device (Mobil-O-Graph PWA Monitor, I.E.M. GmbH, Stolberg, Germany) at baseline (T0), 4 weeks (T1), and 8 weeks (T2). Three consecutive measurements were taken at one-minute intervals, with the average serving as the office BP values. Central BP was recorded 30 s after the final office BP measurement, using appropriately sized cuffs, as per the 2023 European Society of Hypertension (ESH) guidelines [5].

All participants underwent a 12-lead resting ECG to assess HR and other parameters, including PR interval, QRS duration, QT and QTc intervals; also, markers of cardiac HMOD, namely indices of left ventricular hypertrophy (LVH), were assessed, according to 2023 ESH guidelines [5]. ECG assessments were performed at baseline (T0), 4 weeks (T1), and 8 weeks (T2).

In a subset of five patients per group, 24 h ambulatory BP monitoring (ABPM) and echocardiography was conducted at baseline and at 8 weeks to provide detailed insights into BP variations.

ABPM was performed by an oscillometric device (Spacelabs 90,207 On-track, Spacelabs Inc., Redmond, WA, USA). The device was set in the Hypertension Unit after completion of the office BP measurements and the ABPM was started at about 10:00 AM. Automatic BP readings were obtained every 15 min during the day-time period (from 6:00 AM to 22:00 PM) and every 30 min during the night-time period (from 22:00 PM to 6:00 AM) over the 24 h. Each patient was instructed not to alter their usual schedule during the monitoring period, asked to avoid unusual or extreme physical activities, and to maintain the arm still during the BP measurement. Average values for the 24 h, day-time and night-time systolic and diastolic BP levels and heart rate were extracted. For all BP measurements, appropriate cuff sizes were applied, depending on arm circumference of individual patients (small 6–11 cm, small/medium 10–19 cm, medium 18–26 cm, large 22–32 cm and extra-large 33–47 cm), according to the recommendations from the 2023 ESH guidelines [5].

Transthoracic echocardiography was performed on a subset of participants in the left lateral decubitus position using a Philips EPIQ 7 ultrasound system. Evaluations followed established guidelines and were conducted by a single experienced operator to ensure consistency [13]. Measurements included left atrial dimensions, left ventricular (LV) internal dimensions (end-diastole and end-systole), interventricular septal thickness (IVSd), posterior wall thickness (PWTd), and ejection fraction, which was calculated using the Teichholz method. LV function and geometry were assessed. Participants were classified into four geometric patterns: normal geometry, concentric remodeling, concentric hypertrophy, and eccentric hypertrophy [14]. Diastolic function was assessed through transmitral Doppler and Tissue Doppler Imaging (TDI), focusing on parameters such as E/A ratio, deceleration time (DTE), isovolumic relaxation time (IVRT), and the E/e’ ratio. Epicardial adipose tissue thickness was quantified at end-diastole from parasternal long-axis echocardiographic images [15]. Measurements were averaged over three cardiac cycles, with the mean value used for analysis.

After an 8 h fasting period, samples were drawn to assess metabolic markers, including FPG, insulin levels, TOT-C, high-density lipoprotein cholesterol (HDL-C), LDL-C, TG, serum creatinine (and estimated glomerular filtration rate [eGFR]), GOT, and GPT. These tests were conducted at baseline and after 8 weeks. All biochemical analyses, including glucose and lipid profile assessments, were conducted at the Sant’Andrea University Hospital’s certified clinical laboratory. Standardized enzymatic colorimetric assays were employed, following protocols routinely used in clinical research. The Sant’Andrea Hospital UOC laboratory of clinical analysis and biochemistry is designed to ensure the highest quality in diagnostic, therapeutic, and personalized procedure [16,17].

A validated standardized questionnaire, the Greene Climacteric Scale (GCS), was administered to all patients, to evaluate their climacteric symptoms, ensuring a standardized and reliable assessment of clinical conditions before and after treatment. This questionnaire, which independently measured psychological, anxiety, depression, somatic, and vasomotor symptoms [18], was administered at baseline and after 8 weeks.

This study was conducted in accordance with the principles outlined in the Declaration of Helsinki, the International Conference on Harmonisation (ICH) Guidelines for Good Clinical Practice, and the regulations governing clinical trials in Indonesia. Informed written consent was obtained from all participants.

### 2.3. Primary Outcome

The primary outcome of the study was the reduction in office brachial office systolic BP after 8 weeks of treatment with NS compared to that reported in the control group.

### 2.4. Secondary Outcome

Secondary outcomes included (1) reduction in brachial office diastolic BP; (2) reduction in central systolic and diastolic BP; and (3) reduction in lipid parameters, including TOT-C, LDL-C, and triglycerides. Additional outcomes included changes from baseline to 8 weeks in HDL-C levels, FPG, insulin, body weight, and menopausal symptoms, providing a comprehensive assessment of the intervention’s impact on cardiovascular and metabolic health.

### 2.5. Statistical Analysis

The Shapiro–Wilk test was employed to assess whether continuous variables followed a normal distribution. Variables with a normal distribution were expressed as mean ± standard deviation (SD), whereas non-normally distributed data were summarized as median with interquartile range (IQR). Statistical analyses were performed to evaluate the distribution and differences across groups. For normally distributed continuous variables, comparisons were conducted using analysis of variance (ANOVA) for comparisons among multiple groups. To ensure accuracy in detecting changes within groups over time, the Bonferroni post hoc test was applied following significant ANOVA results, correcting for multiple comparisons. Due to sample size constraints/data structure, we opted for separate one-way ANOVAs at each time point to evaluate group differences independently. Categorical variables were analyzed using chi-square tests to assess associations between groups.

A logistic regression model was used to investigate the influence of specific baseline characteristics on the primary outcome (defined as significant arterial pressure reduction). Independent variables included age (<55 years vs. ≥55 years), time since menopause (<5 years vs. ≥5 years), and heart rate (>70 bpm vs. ≤70 bpm). The decision to dichotomize these variables was made to facilitate statistical modeling and to improve the interpretability of odds ratios (ORs) in the logistic regression, in accordance with latest guidelines [5]. The regression model reported coefficients, odds ratios (OR), 95% confidence intervals (CI), and *p*-values to identify significant predictors of treatment response. Since this was an observational study (rather than a randomized controlled trial), logistic regression was used, as it is a powerful tool to adjust for confounding variables that could impact BP reduction or metabolic changes.

Given the fact that this was a pilot study, we did not apply a formal sample size calculation. Statistical significance was defined as *p* < 0.05 for all tests. Analyses were conducted using Stata version 16.0 (StataCorp, College Station, TX, USA) for univariate and multivariate analyses, and Python Version 3.8 (statsmodels library) for paired *t*-tests and regression modeling. This approach allowed for the evaluation of both changes within groups and the identification of predictors influencing treatment efficacy.

## 3. Results

**Figure 1:**
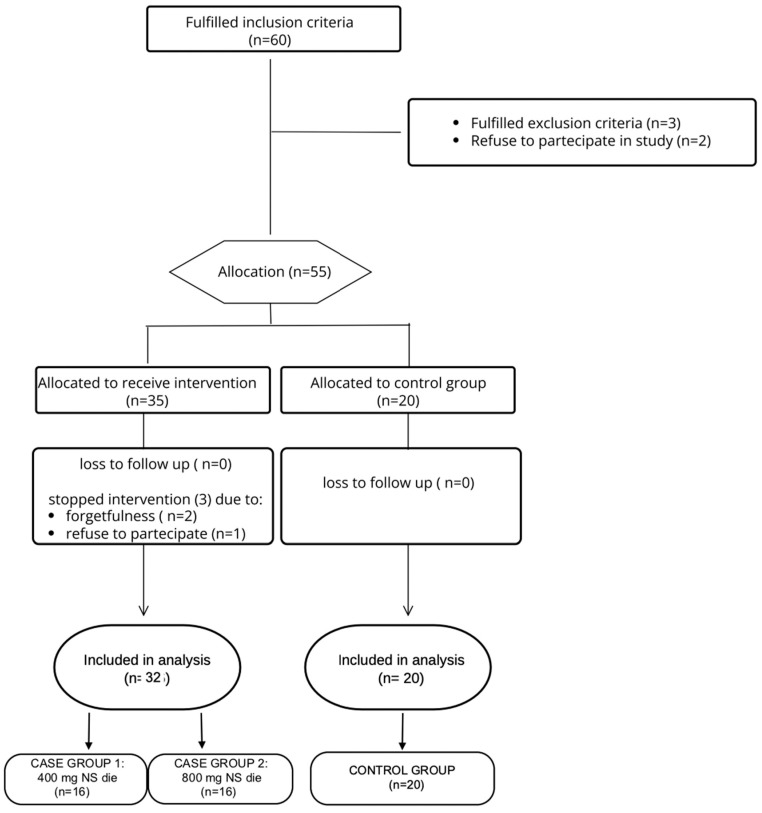
Study Flowchart. This figure outlines the design of the study, detailing participant enrollment, allocation into groups, the intervention protocols, and evaluation time points across the study period.

A total of 60 women with essential uncomplicated hypertension initially met the inclusion criteria. However, five patients (8.3%) declined to participate. Of the remaining 55 participants, 3 subjects (5.5%) from the NS group discontinued the study due to forgetfulness (2 participants; 3.6%) and voluntary withdrawal (1 participant; 1.8%). Thus, an overall sample of 52 patients (86.7%) completed the study, comprising 32 participants in the NS group (61.5%) and 20 participants in the control group (38.5%), as shown in Figure 1.

Participants who completed the study confirmed adherence to the assigned medication dosages throughout the study period. As shown in Table 1, the baseline characteristics of the study groups were comparable across all evaluated prognostic factors.

At baseline (T0), there were no significant differences between the groups in terms of cardiometabolic risk factors and background therapies, confirming that the groups were well-matched prior to the intervention.

As shown in Table 2, both brachial and central office systolic/diastolic BP showed similar values across the groups without statistical significance. Similarly, pulse pressure and other vascular parameters related to arterial stiffness, such as pulse wave velocity (PWV) and augmentation index (AI@75%), also revealed no significant differences among groups at baseline observation.

After 4 weeks (T1) of treatment, the data indicated trends of BP reductions across the groups, even though not statistically significant (*p* > 0.05) except for heart rate (*p* = 0.040), as reported in Table 3.

**Figure 2:**
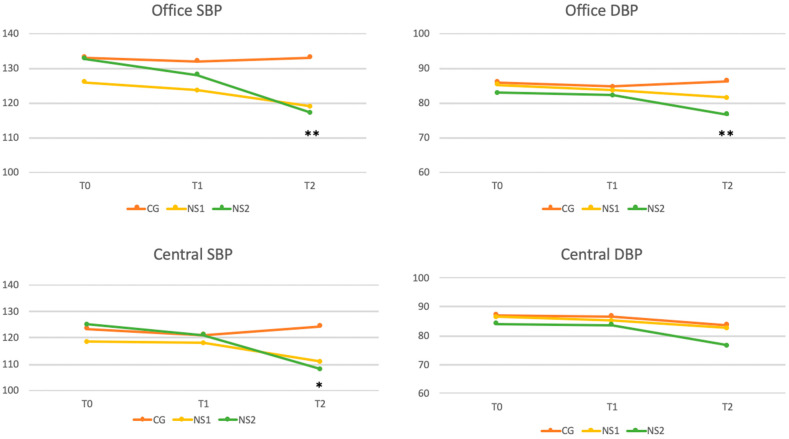
Blood Pressure Changes Over Time. This figure illustrates the changes in brachial and central blood pressures across the study period, highlighting differences between baseline, 4 weeks, and 8 weeks. The statistical significance of the observed changes is marked, with * indicating *p* < 0.05 and ** indicating *p* < 0.01.

At the end of the follow-up period, after eight weeks (T2) of observation compared to baseline values, statistically significant differences were for systolic (*p* = 0.002) and diastolic (*p* = 0.008) brachial BP and pulse pressure (*p* = 0.048). Moreover, central systolic (*p* = 0.016) BP values were statistically significantly reduced compared with the beginning. On the other hand, diastolic (*p* = 0.523) BP and pulse pressure (*p* = 0.051) exhibited a slight reduction but did not reach statistical significance in some comparisons (Figure 2).

**Figure 3:**
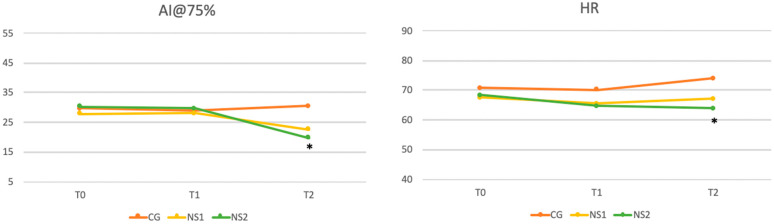
Vascular Parameters and Heart Rate. This figure visualizes the trends in arterial stiffness and vascular health, as measured by PWV and augmentation index, alongside changes in heart rate over the course of the study. These data underscore the effects of the intervention on vascular and autonomic regulation. The statistical significance of the observed changes is marked, with * indicating *p* < 0.05.

As shown in Figure 3, significant reductions in AI@75% were observed at final observation compared to baseline values (*p* = 0.015), whereas no significant differences were found for PWV (*p* = 0.139). On the other hand, HR showed slight but significant increase at final observation (*p* = 0.021).

To further investigate the significant differences identified by ANOVA, a post hoc Bonferroni correction was applied to pairwise comparisons between the three groups. Significant reductions in SBP for both NS1 and NS2 compared to CG were reported. The CG-NS1 comparison yielded a corrected *p*-value of 0.008, while CG-NS2 demonstrated a stronger difference (*p* = 0.0006). No significant difference was observed between NS1 and NS2.

A significant reduction in diastolic BP was also observed between CG and NS2 (*p* = 0.00016), while no significant differences were found between CG and NS1 or NS1 and NS2. Pulse pressure comparisons revealed significant differences between CG and NS1 (*p* = 0.03) and between NS1 and NS2 (*p* = 0.0007). The analysis demonstrated significant differences in Central SBP between CG and both NS1 (*p* = 0.023) and NS2 (*p* = 0.0009), with no significant difference between NS1 and NS2. A significant reduction in heart rate was observed between CG and NS2 (*p* = 0.005). Finally, AI@75%, resulted significantly reduced in NS2 compared to CG (*p* = 0.018), with no significant differences for other group comparisons. All these comparisons are reported in Table 4.

At baseline, there were no statistically significant differences between the three groups for all lipid parameters, as indicated by *p*-values above 0.05 as reported in Table 1. At T2, significant reductions in TOT-C and LDL-C levels were reported, particularly in NS2 compared to the control group. Other lipid parameters exhibited trends of improvement, though without achieving statistical significance, as reported in Table 5.

Furthermore, significant reductions in body weight and BMI in the NS2 group were observed over the study period. The mean weight in the NS2 group decreased from 62.30 ± 5.92 kg to 58.01 ± 5.09 kg at T1 towards 57.09 ± 6.08 kg at T2 (*p* = 0.044). Similarly, BMI showed a significant decrease from 24.39 ± 1.32 kg/m^2^ to 23.21 ± 2.39 kg/m^2^ at T1 towards 22.08 ± 1.52 at T2 (*p* = 0.042). These results suggest a substantial improvement in weight and BMI in the NS2 group, complementing the primary outcomes of the intervention.

**Figure 4:**
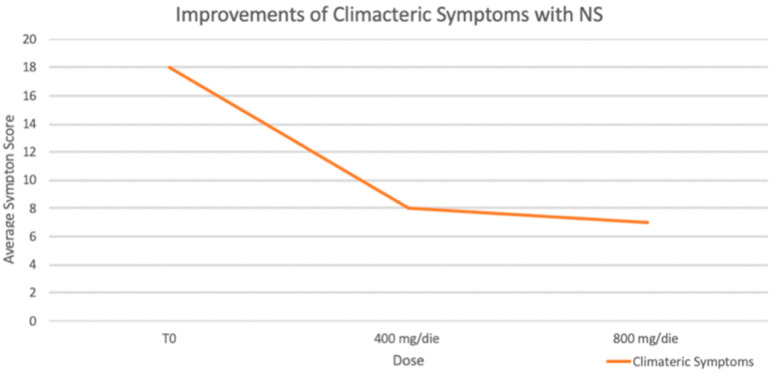
Climacteric Symptoms. Significant improvement in climacteric symptoms as measured by the Greene Climacteric Scale were reported.

Moreover, an improvement in climacteric symptoms (GCS) was reported by patients in both treatment groups. As shown in Figure 4, significant improvement in climacteric symptoms, as measured by the Greene Climacteric Scale, was observed during the observation. With a maximum possible score of 60, symptom severity significantly decreased from a baseline average of 18 to 8 with a 400 mg/day dose and further to 7 with an 800 mg/day dose (* *p* < 0.05 for both reductions).

In the optimized logistic regression analysis, we evaluated the predictors of response to the intervention, defined as a significant reduction in systolic office BP. The model included three independent variables: age (<55 years), time since menopause (<5 years), and heart rate (>70 bpm). The analysis yielded highly significant results, indicating that all three factors are strong predictors of response to the intervention.

The logistic regression model demonstrated excellent predictive capability, with a pseudo R-squared value of 0.5559. The model was globally significant (LLR *p*-value: 1.308 × 10^−16^).

- Age (<55 years): Being younger was significantly associated with a higher likelihood of response (coefficient 2.88, *p* < 0.001). Specifically, individuals aged < 55 years had approximately 17 times higher odds of responding to the treatment compared to those aged ≥ 55 years.
- Time Since Menopause (<5 years): A shorter duration since menopause was a strong predictor of response (coefficient 4.41, *p* < 0.001). Women who had been in menopause for less than 5 years exhibited an approximately 82-fold increase in the odds of response compared to those with a longer duration since menopause.
- Heart Rate (>70 bpm): A higher baseline HR was also significantly associated with response to the treatment (coefficient 5.16, *p* < 0.001). Participants with HR > 70 bpm had approximately 174 times higher odds of responding compared to those with HR ≤ 70 bpm.

## 4. Discussion

The findings of our study align with previous evidence, demonstrating the beneficial effects of NS on BP reduction and CVD protection [7]. In line with the results reported by Dehkordi and colleagues, who investigated 108 mild hypertensive patients receiving 100 mg and 200 mg of NS extract twice daily for 8 weeks, we observed significant reductions in both systolic and diastolic BP (*p* < 0.01 for all) compared to the control group [19]. Our findings reinforce the hypothesis of NS as a potential complementary therapeutic option for hypertension management.

Indeed, the observed effects during the study can be attributed to the supplementation rather than changes in the background antihypertensive therapy. Specifically, it is important to note that the antihypertensive therapy of participants remained unchanged throughout the study, ensuring that any observed improvements in blood pressure and metabolic parameters were not due to modifications in pharmacological treatment. Therefore, while potential interactions between *Nigella sativa* (NS) and antihypertensive drugs cannot be entirely ruled out, the consistent medication regimen across groups suggests that the effects observed are primarily attributable to NS supplementation. These treatments included ACE inhibitors, diuretics, and beta-blockers. At baseline (time 0), the patients’ average blood pressure was not optimally controlled, with values classified as high-normal (135/85 mmHg). This approach allowed us to isolate the potential impact of the supplement, while ensuring that the foundational pharmacological management of hypertension remained consistent throughout the study period.

Interestingly, our results show that the effects of the supplement are dose dependent. While brachial and central systolic BP decreased significantly at both dosages, diastolic BP reductions were only observed with the higher dosage (800 mg/day) (Table 4 and Figure 2) This highlights the importance of optimizing the dosage of NS, to achieve better cardiometabolic benefits. At the 4-week time point (T1), significant reductions were primarily observed in HR, suggesting that HR may be one of the first parameters to respond to the intervention. It should also be noted that the full spectrum of benefits, including reductions in systolic and diastolic BP, became evident only at 8 weeks. These findings emphasize the need for a treatment duration of at least 8 weeks to achieve the therapeutic effects, particularly for metabolic and vascular parameters.

The individualized and tailored approach to therapy emerges as a crucial consideration in this context. While lower doses (400 mg/day) were effective in improving SBP the higher dose (800 mg/day) provided more pronounced metabolic and vascular benefits, including improvements in arterial stiffness (AI@75%), HR, weight, BMI, and lipid profile (C-LDL). This supports the notion that patient stratification based on baseline characteristics, such as age, menopausal status, and baseline HR, may optimize treatment responses. As observed in our analysis, individuals with younger age, more recent menopausal transition, and higher baseline HR experienced more significant improvements, underscoring the importance of precision medicine.

Our study further revealed that the higher dosage of NS (800 mg/day) induced significant reductions in body weight and BMI, as well as improvements in the lipid profile compared to the control group. These effects may be attributed, at least in part, to the supplement’s ability to inhibit HMG-CoA reductase, thereby reducing endogenous cholesterol synthesis and promoting LDL receptor upregulation, as suggested by previous studies [6]. Such mechanisms may contribute to improved lipid metabolism and cardiovascular risk reduction. Moreover, we observed improvements in climacteric symptoms, as reported through validated questionnaires. Notably, these improvements were observed early and even at the lower dosage (400 mg/day). These findings suggest that lower dosages may still provide psychological and autonomic benefits, as well as reductions in systolic BP, which could guide clinical decisions for specific patient phenotypes, especially those experiencing menopausal symptoms and early signs of arterial hypertension [18].

Finally, no side effects or adverse reactions, including renal, hepatic, and patient-reported adverse events, were reported during the entire observation, thus confirming the safety and good tolerability of NS supplementation in postmenopausal women with essential hypertension.

This study is not without limitations. First, the relatively small sample size may limit the generalizability of the findings. While our results suggest a potential role for *Nigella sativa* supplementation in improving blood pressure and metabolic parameters, larger, well-powered studies with more diverse populations are necessary to confirm these effects and assess long-term outcomes. Second, the study design allowed participants, in agreement with their clinicians, to choose their respective groups. While this approach reflects a real-world clinical setting, it introduces selection bias, as individual preferences, pre-existing expectations, or health conditions could have influenced group allocation. Third, awareness of being in a treatment group (or a control group) may have influenced participant behavior, leading to expectancy effects that could impact subjective and physiological outcomes. A randomized design in future studies would help to mitigate these limitations. Moreover, environmental factors, such as climatic variations, may have influenced the outcomes. Finally, while antihypertensive therapies were individualized based on hypertensive phenotypes, they were not uniform across the cohort. As such, it is not possible to definitively determine whether the observed effects of the supplement might have been influenced, either positively or negatively, by interactions with specific pharmacological treatments. Future studies should consider stratifying patients based on their background therapies or standardizing the antihypertensive regimen to better assess the independent effect of the supplement. These factors should be carefully controlled or accounted for in subsequent studies to ensure the robustness of the findings. Finally, although the relatively small sample size may impact the generalizability of our findings, other factors such as population characteristics, baseline metabolic variability, and individual dietary habits may also influence treatment responsiveness. Additionally, the study’s 8-week duration limits the ability to assess long-term effects, warranting further research to explore the sustained impact of *Nigella sativa* supplementation on cardiometabolic health.

## 5. Conclusions

Our study highlights the potential of NS as a versatile therapeutic option for cardiometabolic, and climacteric health. However, our findings should be interpreted with caution due to certain limitations. The relatively small sample size, the non-randomized design, the awareness of being in a treatment group may have influenced behavioral and physiological responses. Despite these limitations, our study provides important preliminary evidence supporting the potential benefits of NS supplementation in hypertensive postmenopausal women. The use of NS associated with conventional antihypertensive medications demonstrated additional antihypertensive effects, along with improvements in arterial stiffness, climacteric symptoms, weight control, and lipid metabolism, while maintaining a favorable tolerability profile. The dose-dependent effects observed in this study, combined with patient-specific responses, emphasize the importance of personalized treatment regimens. However, large-scale, randomized controlled trials are required to confirm these effects and establish their definitive role in hypertension management.

## Figures and Tables

**Table 1:** Baseline Characteristics.

Parameters	CG (*n* = 20)	NS 1 (*n* = 16)	NS2 (*n* = 16)	*p* Value
Age (years)	53.9 ± 3.0	54.2 ± 2.3	52.3 ± 2.4	0.093
BMI (kg/m^2^)	23.9 ± 4.9	23.5 ± 1.8	24.4 ± 1.4	0.212
Smoking (%)	18.7 (%)	19.5 (%)	20.0 (%)	0.994
Dyslipidaemia (%)	56.2 (%)	51.0 (%)	46.7 (%)	0.799
Diabetes Mellitus (%)	3.2 (%)	5.0 (%)	2.0 (%)	0.618
TOT-C (mg/dL)	229.55 ± 42.34	222.00 ± 25.27	223.83 ± 26.67	0.481
HDL-C (mg/dL)	46.55 ± 19.39	63.68 ± 18.61	51.00 ± 10.70	0.082
LDL-C (mg/dL)	148.22 ± 34.05	135.98 ± 28.09	149.78 ± 26.09	0.151
TG (mg/dL)	123.88 ± 48.93	111.62 ± 28.26	115.22 ± 32.58	0.700
FPG (mg/dL)	87.33 ± 11.97	89.25 ± 15.11	88.33 ± 14.22	0.773
Insulin (mg/dL)	8.77 ± 4.75	9.37 ± 5.00	9.49 ± 4.61	0.684
Creatinine (mg/dL)	0.74 ± 0.02	0.77 ± 0.01	0.80 ± 0.02	0.986
Antihypertensive therapy (%)				
Monotherapy (%)	10 (%)	12 (%)	10 (%)	0.851
Dual therapy (%)	61 (%)	64 (%)	67 (%)	
Triple combination therapy (%)	29 (%)	24 (%)	23 (%)	
Low-dose Aspirin (%)	3 (%)	5 (%)	3 (%)	0.382
Lipid lowering therapy (%)	10 (%)	12 (%)	9 (%)	0.853

This table summarizes the demographic and clinical characteristics of the study population. It includes the percentage of female participants, average age, body mass index (BMI), systolic (SBP) and diastolic (DBP) blood pressure, smoking prevalence, and the proportion of participants consuming more than two cups of coffee per day. Distribution of cardiometabolic risk factors, such as dyslipidemia and diabetes mellitus, and other clinical parameters, including lipid, glucose, and serum creatinine levels, are also presented.

**Table 2:** Baseline Blood Pressure and Arterial Parameters.

Baseline	CG (*n* = 20)	NS1 (*n* = 16)	NS2 (*n* = 16)	*p*-Value
Office BP				
SBP (mmHg)	132.9 ± 17.6	125.8 ± 13.3	132.6 ± 15.8	0.415
DBP (mmHg)	85.8 ± 13.4	85.1 ± 9.3	82.9 ± 7.3	0.709
PP (mmHg)	47.1 ± 11.1	40.7 ± 9.6	49.7 ± 15.6	0.153
Central BP				
SBP (mmHg)	123.2 ± 15.9	118.4 ± 13.1	124.9 ± 13.8	0.476
DBP (mmHg)	86.9 ± 12.9	86.4 ± 9.6	84.1 ± 7.7	0.719
PP (mmHg)	36.3 ± 11.3	32.0 ± 10.1	40.9 ± 13.6	0.149
Vascular parameters				
AI@75%	29.6 ± 9.6	27.7 ± 9.8	30.2 ± 9.4	0.774
PWV	8.7 ± 1.2	8.2 ± 1.0	8.6 ± 0.9	0.351
HR (bpm)	70.6 ± 10.9	67.5 ± 5.9	68.3 ± 8.7	0.474

This table provides baseline measurements of both brachial and central blood pressures, including brachial systolic (SBP) and diastolic (DBP) blood pressure. Central systolic (SBP) and diastolic (DBP) pressures are also reported. Pulse pressure (PP), derived as the difference between systolic and diastolic blood pressure, is shown for both brachial and central pressures. Additionally, vascular parameters such as augmentation index (AI@75%), pulse wave velocity (PWV), and heart rate (HR) are included.

**Table 3:** Blood Pressure and Arterial Parameters at 4 Weeks (T1).

4 Weeks (T1)	CG (*n* = 20)	NS1 (*n* = 16)	NS2 (*n* = 16)	*p*-Value
Office BP				
SBP (mmHg)	131.5 ± 15.0	125.5 ± 13.5	128.0 ± 15.5	0.220
DBP (mmHg)	84.5 ± 11.8	83.5 ± 10.5	82.0 ± 9.2	0.340
PP (mmHg)	46.5 ± 10.5	42.0 ± 9.5	46.0 ± 11.0	0.280
Central BP				
SBP (mmHg)	121.0 ± 14.3	118.0 ± 12.8	121.5 ± 13.5	0.190
DBP (mmHg)	86.5 ± 11.0	85.0 ± 10.2	83.5 ± 9.5	0.310
PP (mmHg)	34.5 ± 9.8	33.0 ± 9.2	36.0 ± 10.5	0.250
Vascular parameters				
AI@75%	29.0 ± 8.8	28.0 ± 8.2	29.5 ± 8.5	0.600
PWV	8.5 ± 1.0	8.2 ± 0.9	8.3 ± 0.8	0.450
HR (bpm)	70.0 ± 10.0	65.5 ± 7.5	64.5 ± 7.0	0.040

This table presents the same parameters as in Table 2, measured at the 4-week mark to assess the short-term effects of the intervention on blood pressure and vascular characteristics.

**Table 4:** Blood Pressure and Arterial Parameters at 8 Weeks (T2).

8 Weeks (t2)	CG (*n* = 20)	NS1 (*n* = 16)	NS2 (*n* = 16)	*p*-Value
Office BP				
SBP (mmHg)	133.57 ± 15.37	118.92 ± 13.59 **	117.09 ± 9.01 ***	0.002
DBP (mmHg)	86.14 ± 6.58	81.38 ± 12.59	76.62 ± 6.65 ***	0.008
PP (mmHg)	46.42 ± 13.07	37.56 ± 6.48 *	46.01 ± 6.74 °°	0.048
Central BP				
SBP (mmHg)	124.35 ± 16.86	110.76 ± 13.49 *	108.01 ± 7.25 ***	0.016
DBP (mmHg)	83.64 ± 18.26	82.61 ± 12.69	76.49 ± 8.48	0.523
PP (mmHg)	40.71 ± 19.18	28.15 ± 6.48	31.49 ± 4.47	0.051
Vascular parameters				
AI@75%	30.35 ± 6.92	22.61 ± 12.53	19.62 ± 14.99 *	0.015
PWV	8.22 ± 1.21	7.82 ± 0.81	7.85 ± 0.75	0.139
HR (bpm)	73.92 ± 11.73	67.15 ± 6.14	63.87 ± 6.42 **	0.021

This table displays brachial and central blood pressures, pulse pressure, augmentation index, PWV, and heart rate at the end of the 8-week intervention period, providing insights into the long-term effects of the intervention. The statistical significance of the observed changes between GC and NS1 or NS2 is marked, with * indicating *p* < 0.05, ** indicating *p* < 0.01, and *** indicating *p* < 0.001. The statistical significance of the observed changes between NS1 and NS2 is marked, with °° indicating *p* < 0.01.

**Table 5:** Lipid Profile Changes after 8 Weeks.

Week 8 (T2)	CG (*n* = 20)	NS1 (*n* = 16)	NS2 (*n* = 16)	*p*-Value
TOT-C (mg/dL)	225.41 ± 25.69	212.43 ± 34.08 *	198.31 ± 32.21 **	0.044
HDL-C (mg/dL)	48.73 ± 12.51	61.75 ± 22.69	55.61 ± 15.44	0.124
LDL-C (mg/dL)	151.58 ± 23.37	129.52 ± 34.46	128.17 ± 29.39 **	0.006
TG (mg/dL)	120.93 ± 43.71	110.65 ± 52.84	107.31 ± 32.61	0.556

This table compares the lipid profile after 8 weeks of intervention. Parameters include total cholesterol (TOT-C), high-density lipoprotein cholesterol (HDL-C), low-density lipoprotein cholesterol (LDL-C), and triglycerides (TG), providing a comprehensive view of metabolic changes associated with the intervention. The statistical significance of the observed changes between GC and NS1 or NS2 is marked, with * indicating *p* < 0.05, ** indicating *p* < 0.01.

## Data Availability

The data presented in this study are available on request from the corresponding author, due to privacy issue.
